# An exploratory study of metformin with or without rapamycin as maintenance therapy after induction chemotherapy in patients with metastatic pancreatic adenocarcinoma

**DOI:** 10.18632/oncotarget.27586

**Published:** 2020-05-26

**Authors:** Katherine M. Bever, Erkut H. Borazanci, Elizabeth A. Thompson, Jennifer N. Durham, Kimberly Pinero, Gayle S. Jameson, Amber Vrana, Meizheng Liu, Cara Wilt, Annie A. Wu, Wei Fu, Hao Wang, Yafu Yin, Jeffrey P. Leal, Ana De Jesus-Acosta, Lei Zheng, Daniel A. Laheru, Daniel D. Von Hoff, Elizabeth M. Jaffee, Jonathan D. Powell, Dung T. Le

**Affiliations:** ^1^Department of Oncology, Sidney Kimmel Comprehensive Cancer Center at Johns Hopkins, Baltimore, MD, USA; ^2^Bloomberg~Kimmel Institute for Cancer Immunotherapy at Johns Hopkins, Baltimore, MD, USA; ^3^The Skip Viragh Center for Pancreas Cancer at Johns Hopkins, Baltimore, MD, USA; ^4^Virginia Piper Cancer Center at HonorHealth, Scottsdale, AZ, USA; ^5^Molecular Medicine Division, Translational Genomics Research Institute (TGen), Phoenix, AZ, USA; ^6^Division of Biostatistics and Bioinformatics, Sidney Kimmel Comprehensive Cancer Center at Johns Hopkins, Baltimore, MD, USA; ^7^Department of Nuclear Medicine, Xinhua Hospital, Shanghai Jiao Tong University School of Medicine, Shanghai City, China; ^8^The Russell H. Morgan Department of Radiology and Radiological Science, Johns Hopkins University School of Medicine, Baltimore, MD, USA; ^*^Co-first authors

**Keywords:** pancreatic cancer, mTOR inhibition, maintenance therapy, metformin

## Abstract

Purpose: Metformin combined with the mTOR inhibitor rapamycin showed potential synergistic anti-tumor activity in preclinical studies in pancreatic ductal adenocarcinoma (PDA). This phase 1b study (NCT02048384) was conducted to evaluate the feasibility and activity of metformin +/– rapamycin in the maintenance setting for unselected patients with metastatic PDA (mPDA) treated with chemotherapy.

Materials and Methods: Eligible patients with stable or responding mPDA after ≥ 6 months on chemotherapy were randomized 1:1 to metformin alone (Arm A) or with rapamycin (Arm B), stratified by prior treatment with FOLFIRINOX. Fluorodeoxyglucose (FDG) PET scans and peripheral blood mononuclear cells were obtained for exploratory analyses.

Results: 22 subjects (11 per arm) received treatment per protocol. Median PFS/OS were 3.5 and 13.2 months respectively, with 2 year OS rate of 37%; there were no differences between arms. No responses were observed by RECIST; however, decreases in FDG avidity and/or CA19-9 were observed in several long-term survivors. Treatment related adverse events of Grade ≥ 3 occurred in 0% vs 27% of patients in Arm A vs B and were asymptomatic hematologic or electrolyte abnormalities that were not clinically significant. Improved survival was associated with low baseline neutrophil: lymphocyte ratio, baseline lack of assessable disease by PET, and greater expansion of dendritic cells following treatment.

Conclusions: Metformin +/– rapamycin maintenance for mPDA was well-tolerated and several patients achieved stable disease associated with exceptionally long survival. Further prospective studies are needed to clarify the role of these agents in the maintenance setting and to enhance patient selection for such approaches.

## INTRODUCTION

Pancreatic ductal adenocarcinoma (PDA) is an aggressive cancer with high mortality at all stages and limited treatment options in the advanced setting [1]. The development of highly active multi-agent chemotherapy regimens has led increasingly to prolonged responses on treatment, and treatment duration is sometimes limited not by disease progression but by toxicities [2, 3]. A “maintenance” chemotherapy regimen is often employed [4]; however, a strategy to maintain responses off of chemotherapy is highly desirable. Targeted therapy may be effective in this setting and notably the poly ADP ribose polymerase (PARP) inhibitor olaparib was recently shown to be effective in the maintenance setting for patients with metastatic PDA (mPDA) harboring a germline *BRCA* (g*BRCA*) mutation [5]. In the remaining patients an optimal maintenance strategy remains an unmet need.

Mechanistic/mammalian target of rapamycin (mTOR) is a serine/threonine protein kinase which acts as a signaling node downstream of several oncogenic pathways including KRAS/MEK/ERK and PI3K/Akt, both of which are thought to be relevant drivers in a majority of PDAs [6–9]. mTOR signaling has also been demonstrated to have an important role in T cell differentiation and activation and therefore may mediate the host anti-tumor immune response [10–13]. Despite this, clinical studies have failed to demonstrate efficacy of mTOR inhibitors as a single agent or with chemotherapy in advanced PDA [14–16]. Feedback pathway upregulation likely mediates acquired resistance to these agents, and furthermore, their use is hindered by toxicities, including hyperglycemia, cytopenias, fatigue, and mucositis/stomatitis.

Combining rapamycin with other drugs active in the PI3K/Akt pathway has the potential to overcome resistance and may mitigate toxicities. Metformin is an antidiabetic drug in the biguanide class of agents which inhibits mTOR complex 1 (mTORC1) primarily through AMP-kinase activation [17–20]. A synergistic effect of the combination of metformin with rapamycin was suggested by preclinical studies demonstrating enhanced inhibition of mTOR in a pancreatic cancer cell line and better growth inhibition of pancreatic cancer cells in a xenograft tumor model with the combination than either agent alone [21]. Based on this, we conducted an exploratory study of metformin with or without rapamycin in patients with mPDA in the maintenance setting.

## RESULTS

### Patients and treatments

**Figure 1:**
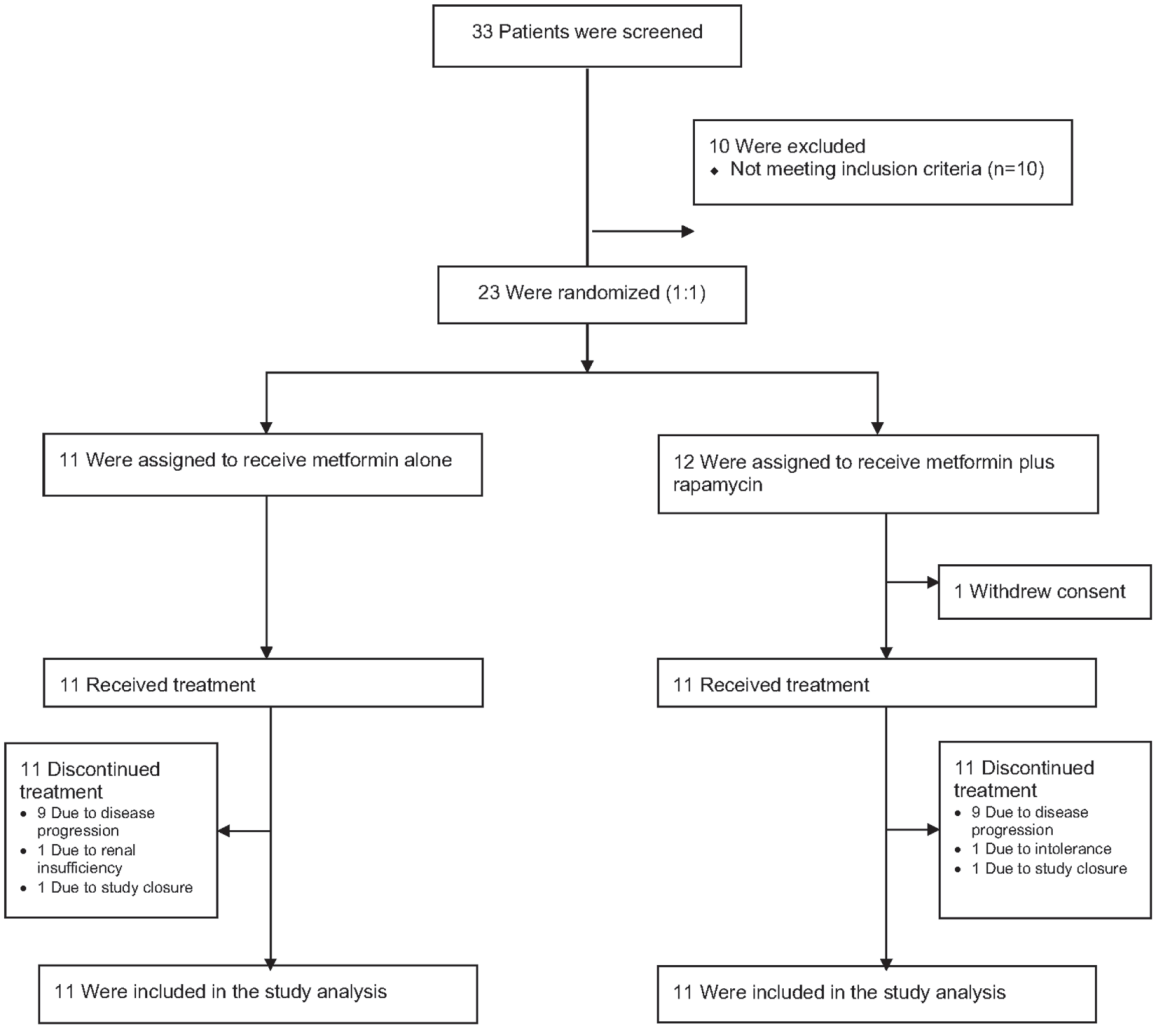
Patient dispositions.

Between June 2014 and December 2017, 23 patients with mPDA were enrolled and underwent randomization, with 11 patients randomly assigned to Arm A and 12 patients randomly assigned to Arm B. One patient assigned to Arm B withdrew consent prior to initiating therapy and was replaced. Thus 22 patients (11 in each arm) were treated per protocol (Figure 1). Patient characteristics were relatively well balanced between the two groups (Table 1). The median time from diagnosis to enrollment was approximately one year, with the majority of patients receiving one or two prior systemic regimens. Most patients previously received a triplet platinum-based regimen, with about half receiving FOLFIRINOX as a prior regimen and several others receiving gemcitabine, nab-paclitaxel and cisplatin (GAC). Prior therapies and results of germline testing and tumor somatic testing are summarized in Supplementary Table 1.

**Table 1:** Baseline patient characteristics

Characteristic	Arm A: Metformin alone	Arm B: Metformin with Rapamycin
	*n* = 11	*n* = 11
***Age-years***		
*median*	58	66
*range*	34–73	52–72
***Sex-no. (%)***		
*female*	6 (55)	4 (36)
*male*	5 (45)	7 (64)
***Race-no. (%)***		
*white*	10 (91)	11 (100)
*black*	1 (9)	0
*other*	0	0
***ECOG performance status-no. (%)^1^***		
0	2 (18)	2 (18)
1	9 (82)	9 (82)
***Tumor location-no. (%)***		
*head*	7 (64)	4 (36)
*body*	2 (18)	4 (36)
*tail*	1 (9)	3 (27)
*unspecified*	1 (9)	0
***Metastatic site-no. (%)***		
*liver only*	6 (55)	3 (27)
*lung only*	0	1 (9)
*other/multiple*	5 (45)	7 (64)
***Histology-no. (%)***		
*well/moderately differentiated*	2 (18)	5 (45)
*poorly differentiated*	7 (64)	2 (18)
*other/unknown*	2 (18)	4 (36)
***Carbohydrate Antigen 19-9-U/mL***		
*median*	46.7	23.4
*range*	10.3–586.5	3.8–218.1
***Prior FOLFIRINOX-no. (%)***		
*yes*	6 (55)	5 (45)
*no*	5 (45)	6 (55)
***Time since first diagnosis-months***		
*median*	11	13
*range*	6.7–43.4	5.6–46.7
***Prior systemic therapies-no. (%)***		
*1*	7 (64)	7 (64)
*2*	2 (18)	4 (36)
*3*	1 (9)	0
≥ *4*	1 (9)	0

^1^ECOG, Eastern Cooperative Oncology Group.

### Safety

Treatment-related adverse events (TRAEs) are summarized in Table 2, according to highest grade observed in an individual subject. In general, treatment was well tolerated. The most frequently observed TRAEs attributed to metformin were gastrointestinal (nausea/vomiting, diarrhea and bloating) and were all mild (CTCAE grade 1–2) in severity. Four patients (Arm A = 2, Arm B = 2) underwent dose reduction of metformin. TRAEs were more prevalent in Arm B, with all patients experiencing at least one TRAE. Three patients (27%) on Arm B experienced ≥ Grade 3 TRAEs, all of which were asymptomatic, including Grade 3 thrombocytopenia in 1 subject, Grade 3 anemia in 1 subject, and Grade 3 hypokalemia and hyponatremia in 1 subject. Dose reduction and/or eventual discontinuation of rapamycin occurred in all patients on Arm B. Subjects were allowed to continue metformin in the case of intolerance to rapamycin requiring discontinuation. One patient in Arm B discontinued study participation due to abdominal pain and redness of hands felt to represent intolerance of study treatments. A patient in Arm A discontinued treatment due to worsening renal insufficiency which was not related to study treatment.

**Table 2:** Treatment-related adverse events

Toxicity-no.	Total	**Arm A (*N* = 11)**		**Arm B (*N* = 11)**	
		Any Grade	G3–4	Any Grade	G3–4
***Any event***	17	6	-	11	3
***Laboratory***					
*platelet count decreased*	6	—	—	6	1
*anemia*	2	—	—	2	1
*neutrophil count decreased*	2	—	—	2	—
*elevated rapamycin level*	2	—	—	2	—
*alkaline phosphatase increased*	1	—	—	1	—
*hypokalemia*	1	—	—	1	1
*hyponatremia*	1	—	—	1	1
***Gastrointestinal***					
*nausea/vomiting*	6	3	—	3	—
*diarrhea, loose stools*	6	2	—	4	—
*abdominal pain*	3	—	—	3	—
*bloating*	3	2	—	1	—
*anorexia*	2	—	—	2	—
*abdominal distension*	1	—	—	1	—
*early satiety*	1	—	—	1	—
*mucositis*	1	—	—	1	—
***Generalized***					
*diaphoresis*	4	2	—	2	—
*chills*	2	1	—	1	—
*edema*	2	—	—	2	—
*fatigue*	2	—	—	2	—
*weight loss*	2	—	—	2	—
*fever*	1	—	—	1	—
*dehydration*	1	—	—	1	—
***Dermatologic***				
*rash*	4	—	—	4	—
*dry skin*	1	—	—	1	—
*pruritis*	1	—	—	1	—
***Respiratory***					
*cough*	1	—	—	1	—
*dyspnea*	1	—	—	1	—
*sore throat*	1	—	—	1	—
*upper respiratory infection*	1	—	—	1	—
***Other***					
*arthralgia*	2	—	—	2	—
*paresthesia*	1	—	—	1	—

^*^Events were counted once for each patient using the highest grading.

### Efficacy

**Figure 2:**
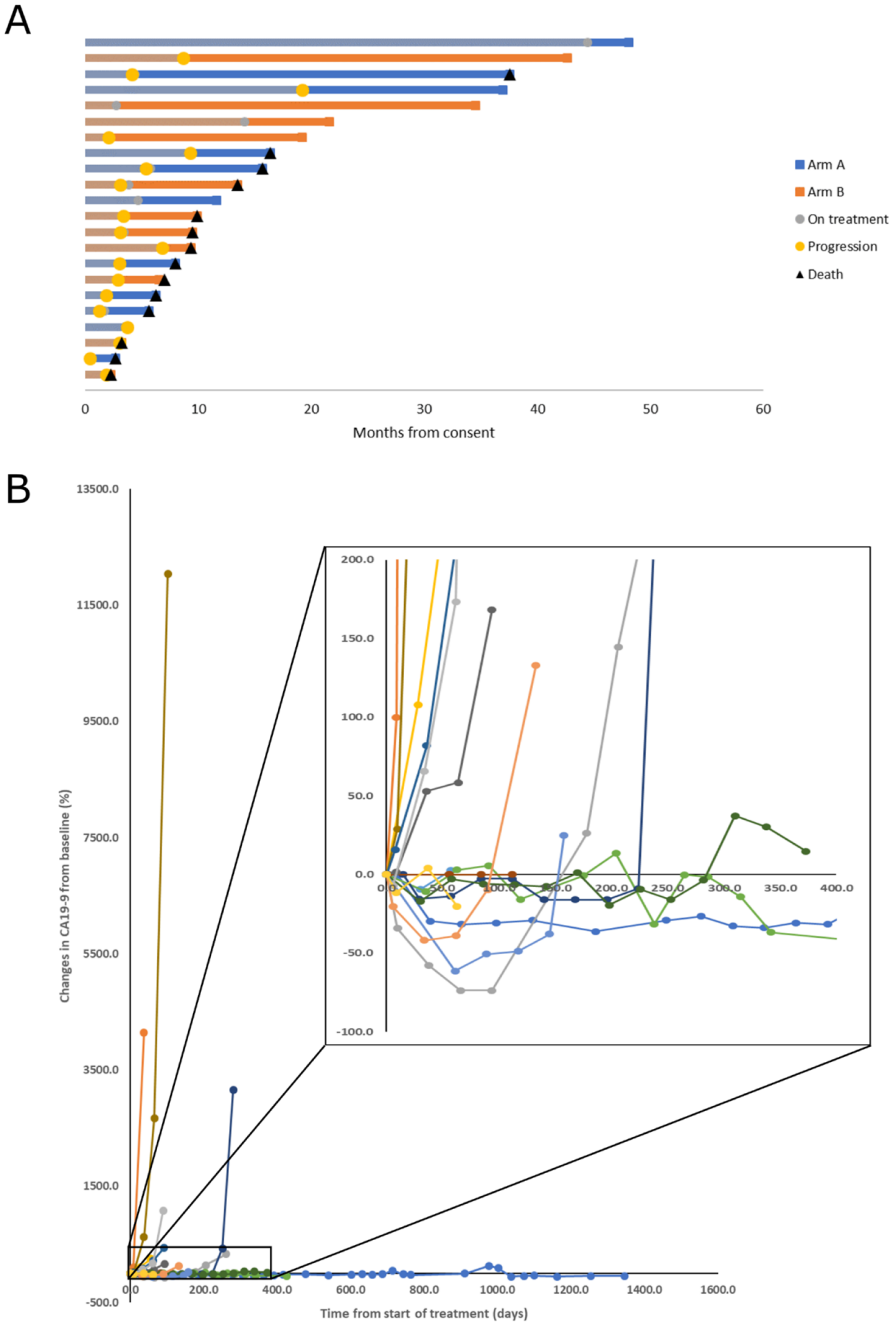
Patient survival and tumor marker kinetics. (**A**) Swimmer plot of patient outcomes demonstrating time on treatment, time to progression, and survival. The end of the bar for subjects still alive indicates time last known alive before censor. (**B**) Spider plot of change from baseline measurement of Carbohydrate Antigen 19-9. Subjects with measurements below the limit of detection at baseline were excluded.

**Figure 3:**
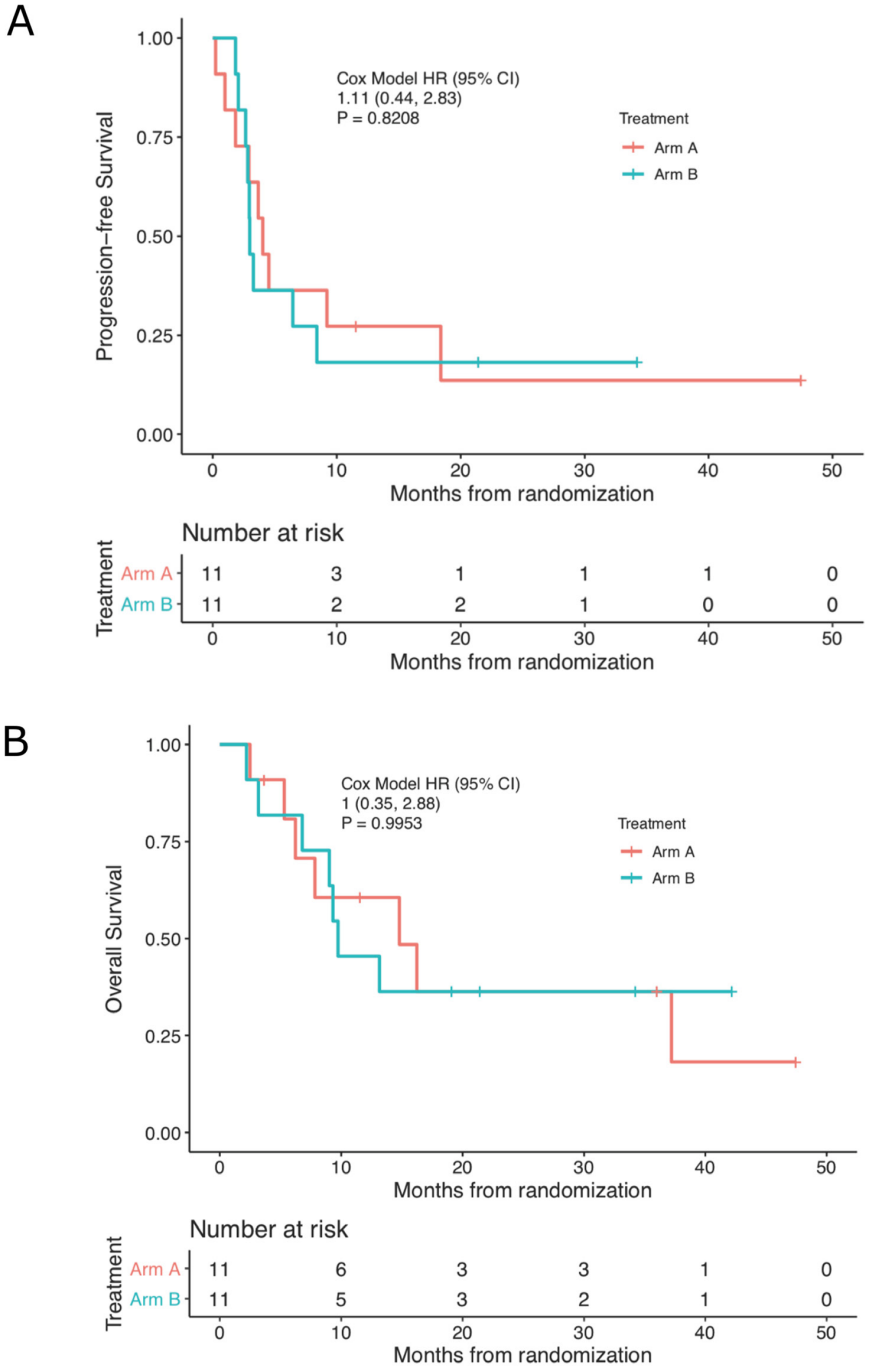
**Kaplan-Meier estimates** of (**A**) progression-free and (**B**) overall survival by treatment cohort.

Time on treatment, time to progression, and time to last follow up or death for each subject are displayed graphically in the Swimmer’s plot in Figure 2A and in table form with details in Supplementary Table 1. The median PFS for the whole cohort, Arm A and Arm B was 3.5 (95% confidence interval (CI): 2.9–9.2), 4.0 (95% CI: 2.9 to not reached (NR)), and 3.0 months (95% CI: 2.8 to NR), respectively. (Figure 3A). There were no deaths occurring before progression. The median OS was 13.2 (95% CI: 7.8 to NR), 14.8 (95% CI: 6.2 to NR), and 9.7 months (95% CI: 9.0 to NR), for the whole cohort, Arm A, and Arm B respectively. (Figure 3B). The 6-, 12-, and 24-month survival rates for the whole cohort were 82%, 53%, and 37% respectively and were similar between treatment arms.

Eight patients in Arm A and 9 patients in Arm B had measurable disease by RECIST 1.1. There were no objective responses observed, and stable disease for a minimum of 8 weeks was achieved in 7 patients on Arm A (88%) and 6 patients on Arm B (67%). Four subjects (2 in Arm A, 2 in Arm B) had unmeasurable CA19-9 at baseline. Individual changes in CA19-9 from baseline measurement in the remaining patients (without knowledge of previous elevation) are plotted in Figure 2B. Five subjects experienced decline in CA19-9 of at least 30%, and 4 of these subjects were among the longest survivors, all with OS > 20 months.

### Correlative analyses

#### PERCIST

Because this was a study in subjects with responding disease, a baseline PET was negative in 6 subjects, and an additional 5 subjects had baseline PET that was not assessable based on the threshold specified. Among subjects with assessable disease at baseline, comparison to on-treatment PET/CT scans revealed no objective metabolic responses; however, two patients with the longest survival had baseline disease which did not meet the threshold for disease assessability but nevertheless had an observed decrease in metabolic activity (21% and 27% decrease respectively) on subsequent PET/CT done after 3 cycles. Baseline PET that was not assessable by PERCIST was correlated with improved OS (HR 0.27, 95% CI 0.08–0.95, *P* = 0.041), but not with PFS (HR 0.47, 95% CI 0.16–1.40, *P* = 0.17).

#### Immunologic and metabolic analysis

Higher baseline neutrophil-to-lymphocyte ratio (NLR) was associated with worse OS (HR 2.82, 95% CI 1.27–6.28, *P* = 0.01) but not PFS. Baseline absolute neutrophil count, absolute lymphocyte count, and platelet count were not associated with clinical outcomes.

**Figure 4:**
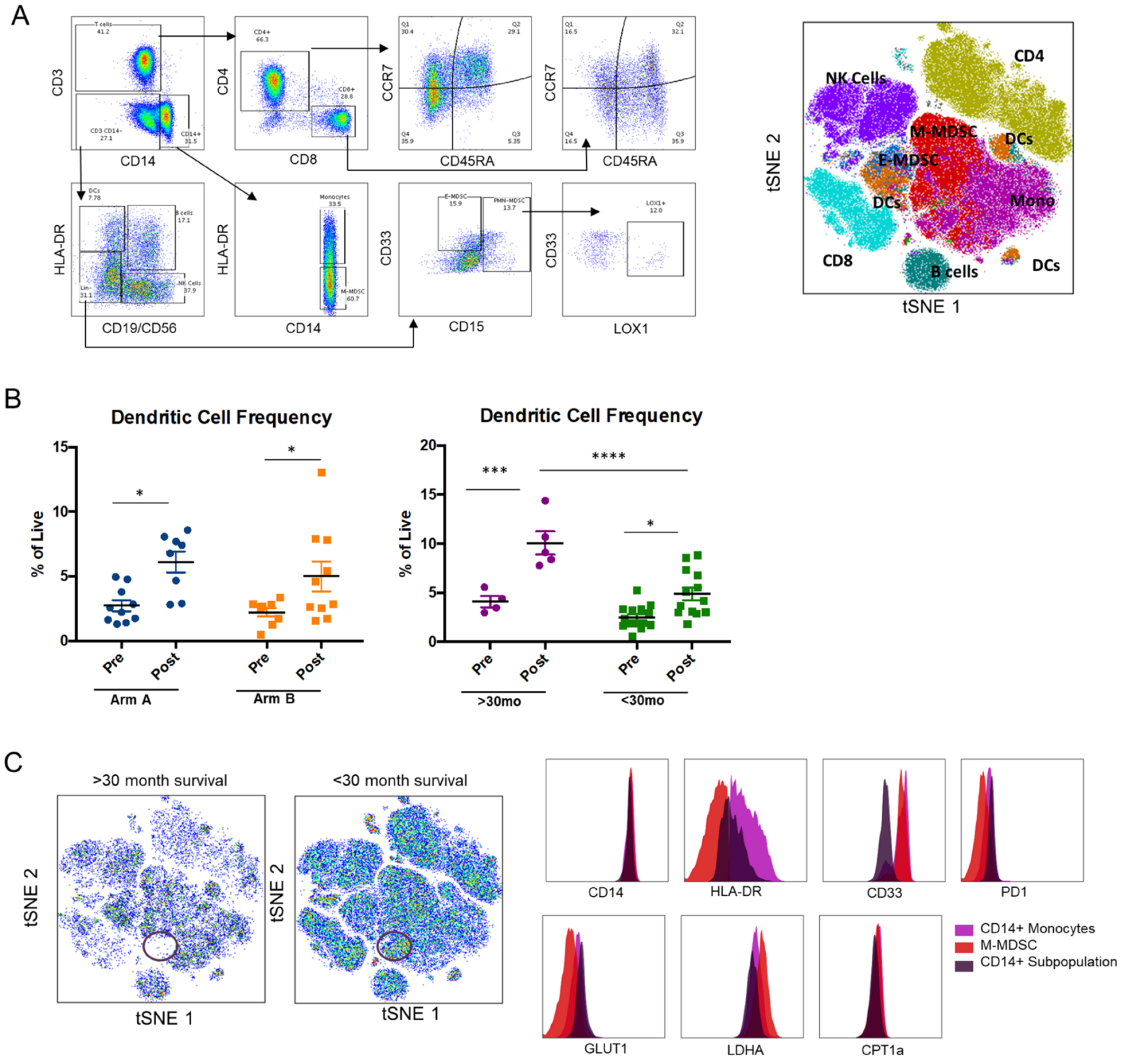
High-dimensional flow cytometric analysis of PBMCs. (**A**) Gating tree demonstrating method for identification of immune cell subsets, and resultant t-distributed stochastic neighbor embedding (t-SNE) plot illustrating distribution of immune cell subsets in PBMCs. (**B**) Dendritic cell frequency (expressed as a percentage of live cells) was determined by this analysis and comparisons of pre- and post-treatment, by treatment arm and by survival are shown in the accompanying dot plots, with the greatest increase noted in long-term survivors. Significance tested using two-way ANOVA with Sidak correction for multiple comparisons. Calculated in Prism version 6.0, ^*^< 0.05, ^**^< 0.01, ^***^< 0.001, ^****^< 0.0001. (**C**) t-SNE plots in long-term survivors versus < 30 month survivors demonstrating relative absence of a subpopulation of CD14+ cells (circled in red) in good prognosis subjects. The relative expression of different markers in this population is shown in comparison to CD14+ monocytes and monocytic MDSCs.

Subpopulations of immune cells were characterized using high dimensional flow cytometric analysis (Figure 4A). No significant effect was observed on T cell numbers or subsets as a result of treatment. This analysis did reveal expansion of dendritic cells in both arms in response to treatment, with higher numbers of dendritic cells post-treatment seen in long-term survivors (surviving > 30 months) (Figure 4B). We attempted to characterize neutrophil subpopulations to explore the aforementioned prognostic association of baseline NLR; however, CD15+ cells were very low across all samples possibly as a result of sample processing.

A population of CD14+ cells was noted to be differentially present in PBMCs of short- versus long-term survivors, both at baseline and on treatment. This population exhibited a unique phenotype of intermediate HLA-DR expression, low CD33, LDHA, and CPT1a, and high PD-1 and GLUT1 expression, and may represent an inhibitory/anti-inflammatory population of monocytes (Figure 4C).

**Figure 5:**
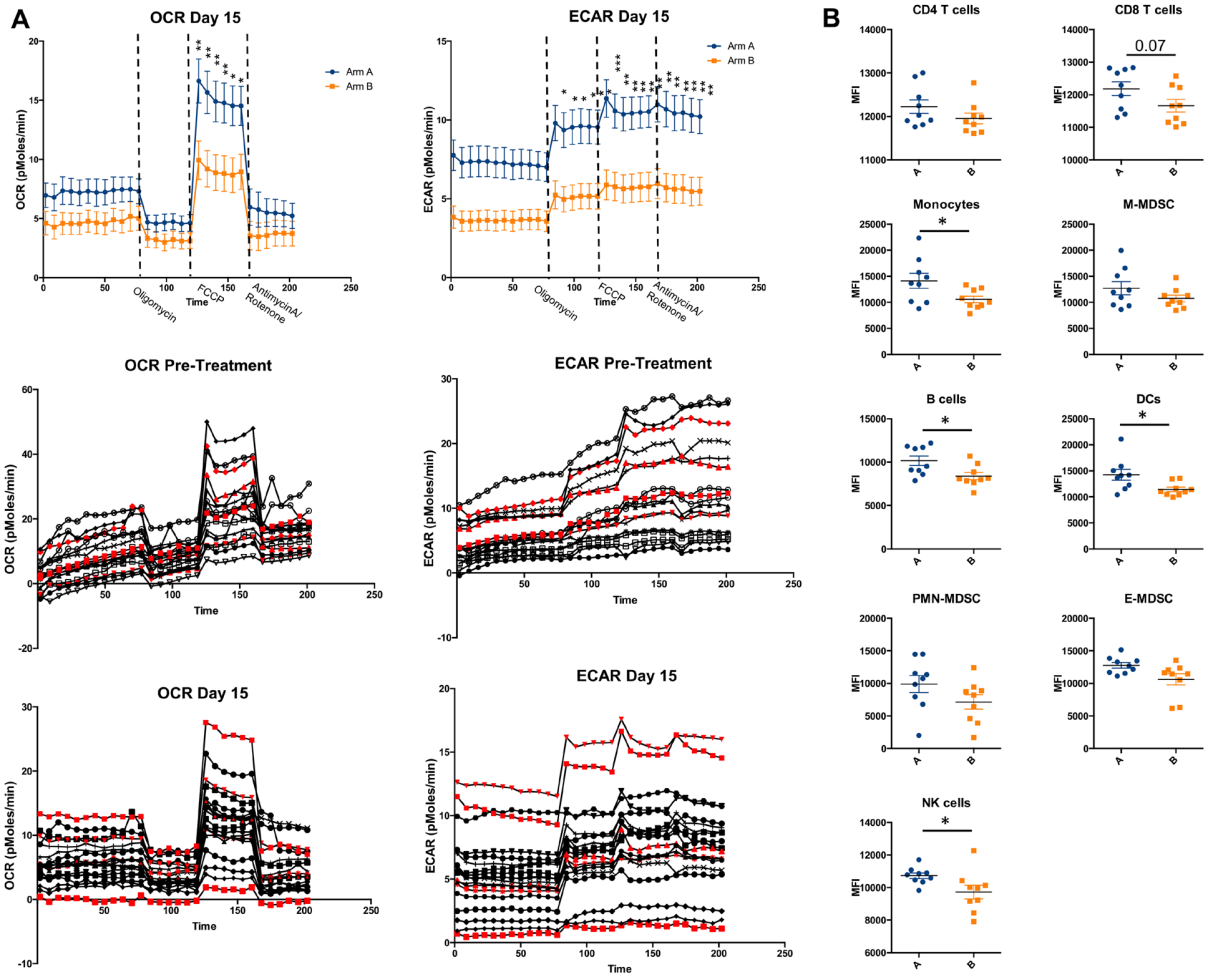
Metabolic analysis of PBMCs. (**A**) Oxygen consumption rate (OCR) and extracellular acidification rate (ECAR) in Day 15 PBMCs samples by treatment arm demonstrates lower metabolic fitness in Arm B. Individual measurements of OCR and ECAR on both pre- and on-treatment samples are shown below with long-term survivors in red demonstrating no clear correlation of outcome with level of metabolic fitness of PBMCs. Significance tested using two-way ANOVA with Sidak correction for multiple comparisons. (**B**) mTOR activity (as measured by pS6 expression) shown in various immune cell subsets, compared between treatment arms. Lower activity was seen in monocytes, B cells, DCs, and NK cells, but not T cells. Significance tested using Mann-Whitney test. Calculated in Prism version 6.0, ^*^< 0.05, ^**^< 0.01, ^***^< 0.001, ^****^< 0.0001.

Metabolic activity of bulk PBMCs was assessed by measuring oxygen consumption rate (OCR) and extracellular acidification rate (ECAR) at baseline and on day 15 specimens. A treatment effect was observed, with both OCR and ECAR significantly lower in subjects in Arm B versus Arm A at day 15; however, there was no appreciable association of either parameter at baseline or on treatment with clinical outcomes (Figure 5A).

Finally, mTOR activity was quantified across immune cell subsets using pS6 expression. Lower mTOR activity was observed in rapamycin-treated subjects in some immune subsets. There were no correlations with regard to mTOR activity in any immune subset and survival (Figure 5B).

## DISCUSSION

To date, mTOR-targeting agents have failed to demonstrate clinical activity in PDA, despite evidence for a significant role of this pathway in tumor progression. In this study, we measured the effect of metformin with or without rapamycin as maintenance therapy in patients with mPDA who achieved a stable disease or response to chemotherapy. Treatment was well tolerated, with a safety profile comparable to what has been reported for the two drugs previously. Dose limiting toxicities were primarily asymptomatic laboratory abnormalities, particularly myelosuppression from rapamycin which may be more prevalent in this patient population who have previous exposure to multi-agent chemotherapy.

We observed a remarkably longer than expected PFS and OS in this poor-prognosis population, including several surviving > 3 years. In contrast to the recently reported phase III POLO trial, in which patients with g*BRCA* mutations were selected for maintenance therapy with olaparib, our patient population was unselected and to our knowledge there have been limited other studies in this population. One such trial of maintenance sunitinib in mPDA reported comparable results, with a median PFS of 3.2 versus 2.0 months for sunitinib versus placebo; there was also a non-significant trend towards longer OS, with a median OS of 10.6 vs 9.2 months and 2-year OS rate of 23% versus 7% respectively [25]. While small numbers, our results, with a median PFS of 3.5 months, median OS of 13.2 months, and 2-year survival rate of 37%, compare favorably with this.

We attempted to answer the question of whether patient selection may account for the long-term survivors observed in our cohort, through examination of patient demographics and treatment history, germline and tumor sequencing (when available) and correlative analysis. As has been extensively reported previously, low NLR did predict for improved outcomes in these patients [26–29], as did lack of FDG-avid disease on baseline PET [30, 31]. In addition, we identified a unique population of CD14+ positive monocytes which were absent in the PBMCs of exceptional survivors, suggesting a potential role for these cells in promoting tumor progression.

Defects in DNA repair pathways may similarly predict for improved outcomes in this setting, as demonstrated in the aforementioned trial of maintenance in patients with mPDA and gBRCA defects, which reported superior outcomes even in the placebo group [5]. Interestingly, while several patients on our study were observed to have germline or somatic mutations or variants of uncertain significance in DNA repair proteins, there was no clear correlation of these mutations with PFS or OS in our trial.

Response assessment was limited in this study in which patients are “debulked” by chemotherapy prior to initiating study treatment. There were no objective responses observed by RECIST or PERCIST; however, we observed declines in CA19-9 and/or decrease in metabolic activity by PERCIST in several subjects, observations which correlated with long-term survival. While not definitive, these “responses” are suggestive that our observations are not due to patient selection alone but potentially due to activity of the agents under study.

Expansion of dendritic cells was observed in both arms in response to treatment and was especially pronounced in long-term survivors. Preclinical studies have suggested a role for mTOR inhibitors in dendritic cell survival and activation that is nuanced and context-dependent [32, 33] and this deserves further study. Alternatively, this may reflect the overall immunologic fitness of the patients with improved outcome.

Although this trial was not powered to detect differences in clinical activity between the treatment arms, we observed decreased metabolic fitness in PBMCs with the addition of rapamycin, in accordance with the known inhibitory function of rapamycin on mTOR activity and the tight link between mTOR activity and upregulation of glycolytic pathways. In further support of this observation, the levels of pS6 were reduced in several immune cell subsets, particularly innate subsets including monocytes and dendritic cells, in the combination arm compared to metformin alone. Importantly, there was no apparent correlation of these observations with clinical outcomes.

A challenge to the application of a maintenance approach is proper patient selection, as clearly not all patients will benefit from continued treatment. To this end, we identified several factors which may be used to select for patients with improved outcomes; however, whether good prognosis patients need any further treatment at all and whether poor prognosis patients will benefit from continued chemotherapy rather than a maintenance approach are not known and additional prospective studies are needed to answer these questions. The availability of newer noninvasive diagnostic testing such as circulating tumor DNA may further clarify this and enhance patient selection.

In conclusion, the administration of metformin with or without rapamycin in patients with mPDA who achieve a response to chemotherapy is well-tolerated and was associated with better than expected overall survival in this study. Additional studies are needed to prospectively evaluate the role of these agents compared to a maintenance chemotherapy or observation only approach.

## MATERIALS AND METHODS

### Patient selection

Patients were eligible for this study if they were ≥ 18 years of age, had histologically- or cytologically-confirmed mPDA, and were previously treated with chemotherapy with stable or responding disease for at least 6 months on the most recent regimen. Two recent scans at least 6 weeks apart were required to confirm disease stability. Other key inclusion criteria were Eastern Cooperative Oncology Group Performance Status of 0 or 1, life expectancy of greater than 12 weeks, and adequate organ and marrow function. Patients were excluded who had brain metastases (unless previously treated and clinically stable for at least 3 months), uncontrolled intercurrent illness, or were taking medications that may interact with rapamycin.

### Study design and endpoints

This was a randomized open-label phase 1b study conducted at the Sidney Kimmel Comprehensive Cancer Center at Johns Hopkins and the Virginia G. Piper Cancer Center at HonorHealth. Enrolled subjects were randomized in a 1:1 ratio to receive metformin 850 mg orally twice daily alone (Arm A), or metformin with rapamycin 4 mg orally daily (Arm B). A minimization randomization approach was used with stratification according to whether or not the patient received prior treatment with FOLFIRINOX, to control for possible differential effects of different chemotherapy regimens. Treatment was interrupted for any event of CTCAE grade 3 or 4 that was considered to be related to treatment. Reduction in the dose of rapamycin was allowed by 1mg increments and a single dose reduction of metformin was permitted to 500 mg twice daily. Discontinuation of rapamycin for intolerance was allowed with continuation of metformin alone. Safety was monitored continuously according to a Bayesian toxicity monitoring rule. Treatment was continued until disease progression, intolerance of study treatments, or study closure, which occurred only after all remaining patients received a minimum of 12 months of treatment.

The study protocol was approved by the institutional review boards of each site and was conducted according to the Declaration of Helsinki and the guidelines for Good Clinical Practice. All patients signed a written informed consent before the conduct of any study procedures and after a full explanation of the study to the patient by the study investigator.

The primary objective of the study was to determine the safety and feasibility of administering metformin with or without rapamycin in subjects with mPDA after disease stabilization on chemotherapy. Secondary objectives were to evaluate fluorodeoxyglucose (FDG) uptake in treated subjects; to measure mammalian target of rapamycin (mTOR) activity in peripheral blood mononuclear cells (PBMCs) of treated subjects; to estimate response rate according to Response Evaluation Criteria in Solid Tumors (RECIST) version 1.1 [22], time to progression (TTP), progression-free survival (PFS), and overall survival (OS); and to measure tumor marker (Carbohydrate Antigen 19-9 (CA19-9)) kinetics.

### Study procedures

Visits occurred every 2 weeks for the first 28-day cycle, then monthly. Complete blood counts and chemistries were obtained with each visit, as well as CA19-9 monthly. For subjects receiving rapamycin, rapamycin levels were obtained monthly starting with cycle 2, and complete blood counts and chemistries were monitored every 2 weeks continuously. Radiologic evaluations were performed at baseline and every 8 weeks throughout treatment. FDG-PET/CT was also performed at baseline, on cycle 1 day 15, and cycle 4 day 1.

### Image analysis

The data analysis of the PET/CT studies was performed using two imaging workstations in tandem – one, a clinical PET/CT system (XD3, Mirada Medical, Inc.), and the other an in-house developed system for PET Response Criteria in Solid Tumors (PERCIST) analysis of PET studies named Auto-PERCIST™ [23, 24]. The lesions of interest were identified by a radiologist using the clinical PET/CT workstation. The Auto-PERCIST™ software was then used to objectively segment and measure the target lesions using methods consistent with a PERCIST analysis: normal reference liver activity was measured, a threshold for disease assessability was determined, and lesion volumes-of-interest (VOIs) were then grown using the highest valued voxel at the target lesion site as the seed coordinate. The resulting lesion VOIs were then measured across multiple metrics which were then saved to a persistent database for subsequent reporting and analysis. The key metrics were sorted and reported in temporal order, with percent changes between timepoints and baseline reported.

### Analysis of PBMCs

Peripheral blood samples were collected into CPT tubes (BD Biosciences) at baseline, on cycle 1 day 15, and cycle 4 day 1, as well as end of treatment. Peripheral blood mononuclear cells (PBMC) were isolated via centrifugation according to the manufacturer’s protocol, washed in media, frozen in fetal bovine serum with 10% DMSO (Sigma-Aldrich), and stored in liquid nitrogen until batch analysis. Upon thawing, cells were stained by flow cytometry or analyzed using the metabolic mito stress test as described below. Antibodies against the following proteins were purchased from Abcam: VDAC1 (20B12AF2), CPT1a (8F6AE9), LDHA (ERP1564), Hexokinase II (EPR20839), Tomm20 (EPR15581-54), GLUT1 (EPR3915). Antibodies against the following proteins were purchased from BD Biosciences: CD8 (RPA-T8), CD3 (SK7), CD33 (WM53), 41BB (4B4-1). Antibodies against the following proteins were purchased from Biolegend: PD1 (EH12.2H7), CD45RA (HI100), CD15 (HI98), CCR7 (G043H7), HLA-DR (L243), CD14 (M5E2), CD19 (SJ25C1), CD56 (5.1H11), CD4 (OKT4), CTLA4 (BNI3), Ki67 (Ki-67), LOX-1 (15C4) and Zombie NIR Fixable Viability Kit. Phospho-S6 ser240/244 (D68F8) was purchased from Cell Signaling Technology. Flow cytometry experiments were performed on a Cytek Aurora and analyzed using FlowJo software (v.10.6; Tree Star).

Metabolic analysis was performed using the Seahorse Cell Mito Stress Test. 150,000 PBMCs were plated per well on poly-d-lysine (50 μg/ml; Sigma-Aldrich)–coated Seahorse XF96 Cell Culture Microplate in XF Assay Medium Modified DMEM supplemented with 25 mM glucose, 2 mM l-glutamine, and 1 mM sodium pyruvate. Experiments were performed using XF 96 Extracellular Flux Analyzer (Agilent Technologies). The following were injected at the indicated time interval: oligomycin (1 μM; Sigma-Aldrich), FCCP (1.5 μM; Sigma-Aldrich), rotenone (2 μM; Cayman Chemical), and antimycin A (1 μM; Sigma-Aldrich).

### Statistical analysis

As the study was exploratory and the analysis primarily descriptive with no formal hypothesis testing planned, a sample size of 22 subjects (11 per treatment arm) was chosen to provide preliminary estimates of the clinical and pharmacodynamic effects of the study drug regimen. Analysis of safety and efficacy endpoints were performed on all subjects who received at least one dose of study drug. Adverse events and toxicity were classified and graded according to the Common Toxicity Criteria for Adverse Events (CTCAE) version 4.0.

Kaplan-Meier curves were used to estimate probabilities of progression-free survival and overall survival at 6, 12 and 24 month, and univariable Cox proportional-hazards model was used to compare differences in time to progression and overall survival between two treatment groups. All statistical tests were two-sided, and *p*-values <.05 were considered significant. All statistical analyses were performed using R version 3.5.3 and GraphPad Prism version 6.0.

## SUPPLEMENTARY MATERIALS


