# Supplementary material for: An exploratory study of metformin with or without rapamycin as maintenance therapy after induction chemotherapy in patients with metastatic pancreatic adenocarcinoma
Source: Oncotarget. 2020 May 26;11(21):1929–41. doi: 10.18632/oncotarget.27586 (PMC7260120; doi:10.18632/oncotarget.27586)
Supplement: Supplementary file 1 [file oncotarget-11-1929-s001.pdf]

An exploratory study of metformin with or without rapamycin as maintenance therapy after induction chemotherapy in patients with metastatic pancreatic adenocarcinoma

SUPPLEMENTARY MATERIALS

Supplementary Table 1: Detailed clinical summary of subjects listed according to survival (longest to shortest)

Subject	Study Arm	Prior Therapy	Time on study (months)	Reason Off Study	Survival (months)	Time to Progression (months)	Time to Death (months)	Germline mutations (Company)	Somatic mutations*** (Company)
9–05	A	FOLFIRINOX	44.46	Study Closure	48.13			None (Color*)	ND/NA
9–08	B	FOLFIRINOX	8.66	Disease Progression	42.71	8.66		None (GeneDx custom panel**)	KRAS, PIK3R, SMAD4 (Foundation)
8–07	A	GAC	4.40	Disease Progression	37.59	4.13	37.59	MUTYH VUS (Invitae)	KRAS, SMAD4, TP53 (Caris)
8–03	A	Gem, GAC	19.21	Disease Progression	36.96	19.21		None (Invitae)	KRAS, TP53 (Caris)
8–06	B	GA, GTX	2.74	Tolerability	34.55			MSH6 VUS (Invitae)	KRAS, TP53 (Caris)
9–13	B	Cy/GVAX/SBRT, FOLFIRINOX	14.12	Study Closure	21.59			None (Color)	KRAS, TP53 (JHH)
9–15	B	Gem/cis, SBRT, GA	2.08	Disease Progression	19.24	2.08		None (Color)	KRAS, IDH2, LEF1, SMAD4 (Perthera)
9–14	A	FOLFIRINOX	9.29	Disease Progression	16.36	9.29	16.36	BRCA1 C64G, BRCA2 VUS (Myriad)	ND/NA
8–04	A	GA, DMOT4039A, FOLFIRI	5.79	Disease Progression	15.70	5.36	15.70	ND/NA	ND/NA
9–17	B	FOLFIRINOX	3.83	Disease Progression	13.52	3.11	13.52	ND/NA	KRAS, RNF43, CDKN2A, PIK3RI, SMAD4, TP53 (Foundation)
9–18	A	FOLFIRINOX, GA, SBRT	4.66	Renal Insufficiency	11.64			ATM VUS (Invitae)	ND/NA
9–01	B	GA	3.40	Disease Progression	9.92	3.40	9.92	ND/NA	ND/NA
8–08	B	GAC/Paricalcitol/ Nivolumab	3.47	Disease Progression	9.49	3.07	9.49	ND/NA	KRAS, TP53, BRCA2 presumed benign variant (Caris)
8–02	B	Gem/5FU/XRT, GAC	6.78	Disease Progression	9.39	6.81	9.39	ND/NA	KRAS, TP53, cMET VUS (Caris)
8–11	B	GA/mabvax	2.98	Disease Progression	6.55	2.88	7.04	PALB2 VUS, RAD51C VUS, SMAD4 VUS (Ambry)	None detected (Guardant 360)
9–09	A	Gem/LDE225, FOLFIRINOX, XRT/ capecitabine, GA, FOLFIRINOX	1.85	Disease Progression	6.28	1.85	6.28	ND/NA	ND/NA
9–02	A	GA/LDE225	3.07	Disease Progression	7.97	3.01	7.97	ND/NA	ND/NA
9–06	A	FOLFIRINOX	1.72	Disease Progression	5.62	1.26	5.62	ND/NA	ND/NA
9–16	A	FOLFIRINOX	3.70	Disease Progression	3.70	3.70		None (Invitae)	BRCA1 (S184fs*50), KRAS, TP53 (Foundation)
8–12	B	FOLFIRINOX	3.21	Disease Progression	3.24	3.04	3.24	None (Invitae)	ND/NA
8–13	A	Gem/cis/paclitaxel	0.40	Disease Progression	2.68	0.40	2.68	None (Ambry)	ND/NA
9–11	B	FOLFIRINOX	1.88	Disease Progression	2.25	1.88	2.25	BRCA1 mut (source unavailable)	ND/NA

Abbreviations: Gem = gemcitabine; cis = cisplatin; GA: gemcitabine/nab-paclitaxel; GAC: gemcitabine/nab-paclitaxel/cisplatin; GTX: gemcitabine/docetaxel/capecitabine; ND/NA: not done/not available; mut: mutation; VUS: variant of uncertain significance; TMB: tumor mutation burden.
Mutations considered pathogenic unless otherwise indicated (VUS).
*This subject underwent Color BRCA 1/2 testing only.
**GeneDx custom panel: ATM, BRCA1, BRCA2, CDK4, CDKN2A, EPCAM, MLH1, MSH2, MSH6, PALB2, PMS2, and PTEN.
***Only classified mutations listed here.
